# The Credibility of Bioethics After the Gaza Genocide

**DOI:** 10.1111/bioe.70052

**Published:** 2025-11-13

**Authors:** Maide Barış, Hossein Dabbagh, Mohammad Sharif Razai, Mehmet İnanç Özekmekçi, Arianne Shahvisi

**Affiliations:** ^1^ Department of Medical History and Ethics Faculty of Medicine, Marmara University İstanbul Türkiye; ^2^ Department of Philosophy Northeastern University London UK; ^3^ Department for Continuing Education University of Oxford UK; ^4^ Primary Care Unit, Department of Public Health and Primary Care University of Cambridge Cambridge UK; ^5^ Department of Political Science and Public Administration, Faculty of Economics and Administrative Sciences Erciyes University Kayseri Türkiye; ^6^ Brighton and Sussex Medical School UK

**Keywords:** bioethics, Gaza–Israel war, genocide, political influence, war

## Abstract

Between October 2023 and January 2025, the Israeli military's sustained attacks on Gaza resulted in an estimated 186,000 deaths and the systematic destruction of healthcare infrastructure. Despite the professed commitment to human dignity, justice, and the minimization of suffering within bioethics, major institutions and scholars in the field have largely remained silent or selectively engaged with the crisis. This paper argues that the Gaza genocide exposes a deeper crisis within bioethics: its growing detachment from urgent, real‐world ethical challenges. Such detachment erodes public trust and raises fundamental questions about the discipline's relevance and credibility. The article interrogates the possible reasons and motivations for the silence and disengagement in the face of genocide in Gaza, and examines the institutional and disciplinary responsibilities that bioethics bears in response to health‐destroying state violence. Framing the inaction as a moral failure with far‐reaching implications, the article proposes alternative routes of ethical engagement and outlines steps toward a more inclusive and responsive bioethics. It calls for the urgent reorientation of the field toward a justice‐driven, politically conscious practice capable of confronting today's most pressing ethical issues.

## 1 Introduction

Between October 2023 and January 2025, Israeli military attacks on Gaza reached a devastating scale, with an estimated 186,000^1^ lives lost directly or indirectly amid widespread violence and destruction [1]. Well‐documented and widely reported strikes by the Israeli army have targeted health facilities, healthcare professionals, and civilian infrastructure in Gaza. These assaults have obliterated essential services, including hospitals, water supply networks, and sanitation facilities, intensifying an already dire and catastrophic situation [2, 3, 4, 5]. Gaza has seen the highest daily death rate of any major 21st‐century war, with more children killed by the Israeli military in a single year than in any recent conflict [6, 7]. Given the scale and nature of such destruction, with repeated attempts to destroy Palestinians’ access to the determinants of health, one might expect urgent concerns and engagement from bioethics institutions. Instead, the discipline has been largely silent [8].

There are many ways to define and demarcate bioethics, but it is widely understood to consist of the study of the normative aspects of life and health, posing and answering ethical questions in relation to individual and public health, healthcare, the life sciences, and the broader biosphere. Central to all the questions tackled within bioethics is the more fundamental challenge of alleviating suffering.

Although the intersection of bioethics and conflict has been addressed in existing literature, it often focuses on military medicine [9, 10], the ethical implications of warfare technologies [11], and the specific dilemmas faced by healthcare workers in conflict zones [12, 13], or remains confined to abstract theoretical analysis [14]. There is little attention to institutional bioethical efforts to support healthcare professionals targeted by state violence, hold perpetrators accountable, or confront the systemic factors that enable such atrocities, revealing a troubling pattern of inconsistency within the field of bioethics.

Therefore, the article questions the relevance and credibility of bioethics and explores the varieties of institutional and disciplinary responsibility bioethics bears in the face of health‐destroying state violence. In this context, the genocide in Gaza is not merely a “test case” for bioethics but a moment of moral reckoning. If bioethics is to remain committed to its widely invoked principles [15]—dignity, justice, and the protection of life—it must confront, condemn, and redress its inaction in the face of genocide. Our aim is not to claim that Gaza must become the singular ethical focus of bioethics but to argue that its erasure, neglect or marginalization signals a deeper failure to deal even‐handedly and proportionately with human suffering, particularly when that suffering is racialized or politicized.

The structure of the article is as follows. In Section 2, we describe the human‐made crisis in Gaza and the responses of global institutions. In Section 3, we discuss the failure of bioethics to meaningfully engage with the genocide in Gaza. Section 4 explores the idea of a reimagined bioethics, while Section 5 suggests some ideas for action within the discipline. Section 6 anticipates objections, and Section 7 concludes the paper.

## 2 The Healthcare Crisis in Gaza and Global Institutions

The healthcare crisis in Gaza has been extensively documented, with repeated calls for urgent action by international institutions, humanitarian agencies, and public health experts. Scholarly contributions from medicine, international law and politics, and genocide studies have pointed to the systematic and deliberate destruction of healthcare institutions and attacks on healthcare workers [16, 17, 18, 19, 20]. These analyses have emphasized that the war in Gaza has not simply degraded the health system; it has made it a central object of military strategy. Hospitals, ambulances, clinics, and water and sanitation systems have been destroyed, healthcare workers have been killed, detained, or displaced, and access to care for civilians has been restricted. This dismantling of health infrastructure has led to what many observers describe as the near‐total collapse of the healthcare system in Gaza [21].

Several authors have argued that these attacks on health institutions and personnel are not incidental but reflect a pattern of targeting that is consistent with genocidal intent [22, 23, 24]. Some have framed the destruction of Gaza's healthcare infrastructure as a violation of international law and medical neutrality, demanding immediate international efforts to restore basic services and safeguard the right to health [25]. Others have invoked the Responsibility to Protect (R2P) doctrine to argue that the international community has a legal and ethical duty to intervene in defence of civilian populations when states fail to uphold humanitarian obligations [26].

Despite these urgent calls, the evidence presented has failed to mobilize meaningful and effective action from global institutions such as the United Nations (UN) and the World Health Organization (WHO) [27]. These institutions carry formal mandates to promote peace, security, and health equity, but their response to Gaza has revealed the structural limitations of international governance. Several interrelated factors contribute to this failure: political obstruction, inconsistent application of mandates, and resource constraints.

First, the UN has often been immobilized by geopolitical divisions, particularly within the Security Council, where veto power by permanent members—including those with strong political ties to Israel—prevents consensus on critical measures such as sanctions, ceasefire resolutions, or peacekeeping interventions [28]. This dynamic renders the UN, as some commentators have described it, “ineffective but indispensable”: essential in principle, but paralyzed in practice [29].

Second, while WHO has issued multiple alerts and updates on the catastrophic healthcare situation in Gaza—including shortages of essential medicines, attacks on hospitals, and restrictions on medical evacuations—its capacity to act is severely limited [30]. Humanitarian corridors have been obstructed, and WHO delegations have at times been denied access to affected regions. Moreover, WHO's reliance on member‐state funding and its lack of enforcement mechanisms diminish its ability to respond proportionately to large‐scale violations of health rights.

Third, even when international institutions issue strong rhetorical condemnations, they often fail to follow through with meaningful protective action. Resolutions and statements have little practical consequence without mechanisms for implementation or accountability [31]. The dissonance between the scale of the crisis and the timidity of institutional responses has eroded public trust not only in these organizations' capacity to act but in their commitment to moral consistency.

The failure of global institutions to uphold their stated principles in Gaza reflects a broader crisis in international humanitarian governance. Despite clear evidence of widespread civilian suffering, targeted attacks on health infrastructure, and violations of international law, the international response has been characterized by inertia, fragmentation, and selectivity. This systemic failure sets the context for the subsequent discussion of the bioethics community, whose own claims to universality, justice, and human dignity demand equally urgent scrutiny [32].

## 3 The Failure of Bioethics to Engage With Israel's Genocide in Gaza

Despite longstanding commitments to justice, dignity, and the protection of life, the bioethics community—encompassing professional associations, academic centers, journals, and thought leaders—has responded to the Gaza genocide with marked silence [33]. While a few individual scholars have published articles expressing concerns [8, 34, 35, 36], these efforts have not been organized attempts to reorient the discipline, nor have the positions expressed been endorsed by influential institutions within the discipline. Taken together, the institutional response has been quiet, hesitant, and incommensurate with the scale of suffering and systematic health‐related harm inflicted on Palestinians.

A scoping review of ten leading bioethics journals found no peer‐reviewed articles addressing the Gaza war between October 2023 and March 2024. Similarly, prominent bioethics blogs and forums—including *The Hastings Bioethics Forum*, *Bioethics Today*, and the *Journal of Medical Ethics* blog—offered sparse coverage, and what appeared was often limited to generalized humanitarian commentary [8]. Authors of this review attribute this disregard to bioethicists' reluctance to engage with politically sensitive issues or to editorial constraints that discourage contributions on ‘controversial’ topics. They argue that this silence erodes the moral foundations of bioethics and raises questions about the discipline's willingness to confront large‐scale violations of medical ethics and human rights [8]. To our knowledge, no major bioethics associations issued public statements or launched initiatives in solidarity with Palestinian civilians, health workers, or institutions.

What could be the possible reasons for the reluctance of bioethical institutions to engage with the situation in Gaza? First, some of this disengagement may stem from institutional caution. In a highly polarized political environment, ethical statements—especially those involving the Israeli‐Palestinian context—are often read as declarations of political alignment rather than principled ethical concern. Scholars may fear being misinterpreted or professionally penalized for appearing to endorse particular ideological stances, especially given that Gaza and Hamas are often conflated, and the latter is a proscribed terrorist organization in the countries whose citizens and institutions dominate bioethics. Others may encounter internal constraints, such as editorial gatekeeping or strategic calculations about funding, reputation, or institutional partnerships [34]. While these are understandable concerns for any institution that depends on external funding, this dependency alone cannot justify disengagement, ethically speaking, since the professional benefits of silence cannot outweigh the benefits of using the moral authority and analytical powers of a respected institution to issue carefully justified ethical condemnation. While individual bioethicists have responded to the genocide, in the absence of institutional amplification, their efforts are liable to have limited impact. Coordinated institutional efforts are much weightier, morally speaking, and are therefore urgently needed if the discipline is to influence the prevailing discourse

Second, the institutional pressures contribute to what we describe not simply as silence but as a pattern of selective visibility, where some forms of suffering are consistently foregrounded, while others are rendered peripheral or invisible [37, 38]. In case of conflict engagement, there seems to be a form of selective engagement with some wars over others, as the discipline has responded in a comparatively uncomplicated way to crises elsewhere [39]. This selective attention becomes particularly apparent when comparing the response to Gaza with responses to other recent conflicts. The Russian invasion of Ukraine was met with rapid and unequivocal condemnation from major bioethics institutions [33]. Within months, *The Hastings Center* had published multiple essays denouncing Russian actions, including support for academic boycotts and embargoes on scientific cooperation [40, 41, 42]. Joseph Fins, former president of the American Society for Bioethics and Humanities (ASBH), urged bioethicists in 2022 to speak out against Russian aggression, warning of “the peril of silence” [43]. Yet ASBH's leadership has remained silent on Gaza, offering no statement or programming in response to the war's impact on civilian health or medical ethics [44]. The American Medical Association (AMA) issued a public statement declaring Russia's conduct to be in violation of “every standard of decency.” While the AMA is not a bioethical institution, reference to a “standard of decency” is an ethical statement. In contrast, when a resolution calling for a ceasefire in Gaza was introduced at the AMA's interim meeting in November 2023, it was rejected on procedural and definitional grounds, with no equivalent expression of concern for the targeted destruction of health systems or medical personnel [44].

The same inconsistency is visible in academic publishing. Jecker et al.‘s 2024 article on the ethics of war and public health identifies several recent crises (e.g., those in Yemen, Syria, and Ukraine) but omits Gaza entirely, despite its clear relevance and escalation by that time.^2^ Moreover, their article fails to cite or engage with a long‐standing body of bioethical literature on Israeli practices in occupied Palestine, including restrictions on access to care, permit systems, and targeting of medical infrastructure [45, 46]. Reticence in the case of Gaza demands explanation.

Third, there may be a misguided attempt to “sit out” the genocide in Gaza to uphold “neutrality” within the discipline. While care and even‐handedness are necessary when developing normative analyses, attempting to adhere to these ideals at the expense of engaging with rapidly unfolding or highly “political” ethical issues will inevitably impact the relevance (in terms of timeliness and impact) of the discipline. And though “neutrality” is often taken to be a virtuous aim, it is not clear whether it is possible, let alone morally commendable, in relation to real, weighty moral issues. Neutrality can and often does serve as an unquestioned excuse for inaction, but as a disciplinary ideal it is inconsistent with the moral imperative for bioethics institutions to intervene when sustained harm is inflicted on vulnerable populations [47].

Feminist and decolonial bioethicists have long argued in their respective traditions that silence and neutrality often mask alignment with dominant power structures, partly by silencing marginalized perspectives [48, 49, 50]. Moreover, they argue, the claim to objectivity often conceals willing, reluctant, or unnoticed complicity with dominant social structures. Institutions within the field have been criticized for their failure to address the racial, cultural, and colonial dimensions of Israel's settler colonial project [8, 33, 51], wherein the lives of Palestinians, particularly children, are often afforded less moral weight within a predominantly Western, Eurocentric bioethical framework. This leads to the effective exclusion of Palestinians from the moral community [52]. In this sense, neutrality is not simply the absence of a position, it is an epistemic stance with tangible consequences. Choosing not to engage with state violence and systemic oppression signals which forms of suffering are deemed intelligible within the moral imagination of the field. Yet recognizing harm is not the same as identifying with those who suffer, nor does it necessarily entail political alignment. The distinction between acknowledging injustice and taking a normative stance is subtle but significant—it marks the boundary between epistemic and political bias, a boundary that bioethics institutions too often fail to acknowledge [53]. Our critique, therefore, does not frame neutrality as deterministically shaping moral discourse, but as participating in epistemic practices that render some lives less visible or audible than others.

As some authors demonstrate, global health discourse has largely failed to name settler colonialism and structural racism as foundational determinants of Palestinian health. This silence, they argue, erases political responsibility and reframes systemic violence as a neutral humanitarian issue [54]. Many bioethicists and bioethics journals operate similarly: by refraining from using or discouraging terms such as “apartheid”, “racism”, and “genocide” in the name of neutrality, they not only align themselves with dominant power structures that strategically reject these terms for the moral weight they command, but they also fail to meet the academic standard of using the correct (which is to say: most accurate and instructive) term for a given phenomenon. There is, therefore, an epistemic element to the moral shortcoming.

The limited engagement of mainstream bioethics with the genocide in Gaza reflects a deeper structural problem in the field—its reliance on frameworks that privilege Western ethical paradigms. As several authors argue, relying on Western methods and applying mainly Western concepts is part of a broader problematic trend of epistemic injustice in global health ethics. Such tendencies marginalize ethical concerns emerging from contexts shaped by colonial histories, geopolitical asymmetries, and racialized violence [53, 55]. In this light, bioethics' silence on Gaza is not merely an oversight but reveals the limits of a discourse that has yet to reckon with global structures of harm. In the face of genocidal violence under a racialized regime of occupation, a purportedly neutral stance is no longer ethically tenable. Bioethics' refusal to confront the ideological foundations of such harm signals not neutrality, but complicity that threatens its credibility.

## 4 The Moral Reckoning of Bioethics

In a 2025 editorial in *Bioethics*, Udo Schüklenk, one of the journal's editors‐in‐chief, responded to criticisms that *Bioethics* journal had remained “too quiet” in the face of grave human rights violations. While he claims to have “strong personal views” he emphasized these are “not significantly informed by [his] bioethics expertise.” Unless ethical questions directly involve issues such as professional duties in military medicine or attacks on healthcare facilities, he argued, they fall outside the discipline's scope [55]. Similar views were voiced at a 2025 conference panel at the University of Oxford entitled “Bioethics and activism: complementary or opposed,” where panellists expressed concerns about endangering their credibility and the field's by speaking outside of their expertise [56]. This seemingly common view is captured by Schüklenk's statement that “My good intentions and strong personal convictions do not make me an expert on war.”

Bioethicists may not be “experts on war,” but they are moral philosophers, scholars of health, and practitioners of justice. When entire health systems and populations are targeted, war becomes a priority area for bioethics. If scarcity of ventilators or vaccinations within a pandemic due to supply chain issues jeopardizes the health of large numbers of people, then supply chain issues become an ethical issue [57, 58], and bioethicists must do their learning to be able to adjudicate the ethical issues. When war jeopardizes the health of large numbers of people, bioethicists must learn what they need to learn about war, which will surely not be everything there is to know, but just enough to set up the normative analysis that is firmly within the scope of the discipline [59]. Even experts on war are not experts on all wars, and few experts on war will be experts on the consequences of war or genocide for the health of those affected, and the associated ethical ramifications. There is a place for bioethicists and if they do not take up that place, important work will be undone. It seems unlikely that any careful argumentation could justify putting caution about disciplinary boundaries ahead of subjecting a health‐destroying genocide to robust bioethical analysis.

There are other ways to do bioethics, even if they are currently more marginal. Kleinman has urged a broader conception of the discipline that is responsive to the lived moral experiences of those affected by systemic violence and political oppression. Reflecting on atrocities such as the genocides in Bosnia, Rwanda, and Kosovo, Kleinman critiques bioethics' failure to address real‐time moral emergencies [60]. Similarly, Jafarey has exposed the pattern of “parachute bioethics,” where ethical analysis is imported from elsewhere and disconnected from the structural realities on the ground [61]. These accounts call for a reimagined bioethics that is grounded, situated, and responsive to lived contexts, such as wars.

Cooley similarly challenges the field's assumptions, advocating a more dynamic, interdisciplinary, and justice‐oriented framework. She calls on bioethicists to move beyond traditional biomedical boundaries and confront contemporary global challenges—war, displacement, environmental collapse—that profoundly affect health yet are often excluded from bioethical discourse [62]. Other critics highlight the discipline's avoidance of politically charged issues and its failure to ground ethical analysis in specific geopolitical contexts [33, 63]. This neglect not only weakens discourse on global justice but also diminishes the role of the humanities in fostering critical activism [51].

Dreger and Baylis articulate a related vision through “Impact Ethics,” which prioritizes public engagement, social accountability, and direct advocacy. Unlike conventional academic bioethics, it is action‐oriented and rejects the idea that commentary alone suffices in moments of ethical crisis. In this model, neutrality ceases to be a virtue when it serves to protect institutions from discomfort while enabling injustice [64].

Furthermore, Lederman invokes Jill Stauffer's concept of *ethical loneliness*—the profound isolation felt when individual suffering is neither acknowledged nor addressed by ethical or institutional systems. He argues that bioethics must confront the moral and institutional abandonment of vulnerable populations, recognizing that ethical loneliness represents not just exclusion but the betrayal of those left unprotected by systems that should have advocated for them [65]. This highlights the urgent need for solidarity‐driven approaches that restore dignity and rebuild trust, preventing bioethics from perpetuating structural neglect.

These critical perspectives are important starting points for evaluating the failures of bioethics during periods of acute crisis, including war and genocide. If scholars and institutions within bioethics cannot offer a clear, robust, unflinching response to the ongoing genocide in Gaza, it is reasonable to question whether they are capable of naming and addressing other injustices faced by marginalized populations. Further, the credibility of bioethics depends on its moral authority. Dereliction of that core duty could do great damage to the discursive and practical influence of the discipline in relation to other important moral issues. What is needed therefore is an urgent disciplinary reckoning and a commitment to global justice, collective dignity, and clear opposition to political and military oppression.

Still, calls for political disengagement in bioethics require critical scrutiny. The notion that the field should transcend political entanglements is hard to sustain in light of its growing engagement with global health inequities, structural racism, and climate injustice—all deeply political in nature [66]. The issue is not politics itself, but the subordination of ethical reasoning to the interests of powerful actors. Ethical engagement in the face of systemic violence is inherently political, but this does not make it partisan; it makes it a matter of real concern. The greater danger lies in evading moral responsibility under the guise of neutrality. What is needed is not a political detachment, but a principled ethics that acknowledges its political stakes and upholds moral clarity.

Neutrality is often taken to be an ethical ideal in many professions and academic disciplines, but is especially, and to some degree understandably, favored in the practice of medicine. A clinician should strive to remain neutral on political matters while providing direct care to patients e.g., a right‐wing patient should receive the care they need even if the clinician is appalled by their politics. (Though it is easy to argue the act of providing care to all is not a politically neutral act.) Clearly, this ideal of neutrality is not, and could not be, upheld in medicine more broadly (e.g. public health, health systems, global health), which is a political enterprise within which political decisions are unavoidable [67]. In bioethics neutrality makes even less sense as a disciplinary ideal: it is a normative enterprise within which positions must be staked out and justified in ways that must be even‐handed but are necessarily never “neutral” with respect to moral and political commitments.

Bioethics must therefore resist pressures that discourage speech, protect privilege, and obscure complicity. A justice‐oriented bioethics demands moral courage: the willingness to name injustice, listen across lines of power, and act in solidarity with the vulnerable. This requires drawing on decolonial insights, centering political responsibility, naming racial violence, and aligning with struggles for collective liberation.

To recap, Gaza has exposed the limits of what bioethics has become—and invites reflection on what it could still become: a discipline marked by moral courage, global solidarity, and rigorous engagement with power. As the next section explores, an engaged bioethics requires more than critique. It requires action.

## 5 Bioethics in Action: Confronting Injustice

While the field of bioethics has historically engaged with health‐related injustice, it has often done so unevenly, particularly in contexts shaped by political violence or state‐perpetrated harm. In the case of Gaza, institutional silence has largely prevailed, but there are notable exceptions. This section reviews some of these outliers as instructive models of what an engaged, justice‐oriented bioethics might look like.

Lederman and Lederman examine the obligations of bioethicists in conflict zones, with a focus on Yemen. They argue that war‐induced health crises demand not only bioethical analysis but direct advocacy and institutional accountability, calling on bioethicists to actively engage in advocacy and policy discussions to protect the right to health in these contexts [68]. Similarly, Fernandes explores the ethical obligations of healthcare professionals to respond to human rights violations and confront the political determinants of health [69]. These authors model a form of bioethics grounded in solidarity, attention to power, and a refusal to remain silent in the face of suffering [8, 33].

In the context of the genocide in Gaza, Shahvisi's editorial in the *Journal of Medical Ethics* offers a compelling example of bioethics in action. She documents the destruction of hospitals, attacks on ambulances, and targeted violence against healthcare workers, framing these as deliberate efforts to produce “health scarcity.” She argues that scarcity of health resources and the determinants of health is a core topic of analysis within bioethics, and those responsible for that scarcity should be held to account by bioethicists. In other words, criticism of Israel's atrocities in Gaza is entirely within scope for the discipline [34].

Building on these calls for “bioethics in action,” we support a model rooted in what Dreger and Baylis term *Impact Ethics*—an approach emphasizing public accountability, direct engagement, and a willingness to challenge dominant power structures [64]. Advocacy, equity, and institutional responsiveness become central responsibilities of the field, not optional extensions of academic work. In situations like Gaza, action is an ethical necessity. This model demands immediate ethical intervention to protect vulnerable populations. It also represents an exemplary framework that invites action to challenge dominant powers [33].

So what might such action look like in practice? A plural, context‐sensitive set of responses is necessary, recognizing that not all institutions share the same capacity or mandate. Nonetheless, each node in the bioethics ecosystem, including but not limited to journals, associations, academic programs, ethics committees, and international networks, can and should contribute to greater moral accountability.

First, academic journals can play a pivotal role by commissioning special issues, rapid‐response commentaries, or reflective pieces on health and conflict. Editorial policies should be revised to include scholarship from and about marginalized, occupied, or war‐affected communities. Journals must also scrutinize how editorial silence or omission may reinforce epistemic injustice.

Second, professional associations and international coalitions of bioethicists bear ethical responsibility to confront global ethical emergencies with coordinated efforts to issue carefully argued moral condemnation where relevant. These bodies can issue public statements, defend academic freedom, and support bioethicists working under politically fraught conditions. They might also host webinars, roundtables, and white papers on urgent themes such as war, settler colonialism, and the ethics of bearing witness. In addition, international networks can amplify voices from the Global South, foster solidarities that resist geopolitical asymmetry, and ensure that transnational collaboration challenges, rather than reproduces, global hierarchies. Global forums must reflect not only institutional diversity but also political and experiential depth.

Third, university‐based bioethics and medical humanities programs should revise curricula to address political determinants of health [70], ethical responsibilities in humanitarian settings, and critical perspectives on neutrality. Partnerships with scholars in conflict‐affected regions should be prioritized not merely for visibility but for reciprocal learning. Emergency fellowships or institutional affiliations can also support displaced researchers whose voices are too often excluded.

Finally, ethics committees and global health review boards must recognize structural violence as ethically significant. While not every committee requires direct representation from conflict‐affected populations, those reviewing research on global health, humanitarian aid, or forced migration should include members with lived or community‐based knowledge. Across all these efforts, representation must be substantive, not symbolic. These roles should not be filled by elite figures standing in for marginalized groups who merely check a box, but by those with genuine insight into the lived struggles of affected populations. Moreover, risk–benefit frameworks should be revised to account for conditions of conflict and occupation, and clinicians navigating moral dilemmas—such as triage, care denial, or forced collaboration—must be supported.

Public trust in the field of bioethics depends not on its detachment, but on its timely engagement with issues of obvious bioethical concern. Therefore, bioethics must engage with crises that threaten health and life on structural, not just clinical, levels. That includes recognizing that health outcomes are shaped not only by access to care, but by the political determinants of health: borders, bombings, blockades, surveillance and dispossession [70]. In Gaza and elsewhere, these are not background variables; they are central ethical facts. Thus, a reimagined bioethics must also learn to listen. It must center the perspectives of those most affected, forge global solidarities, and challenge the geopolitical asymmetries that shape which crises get moral attention and which are ignored.

This vision of bioethics in action is not without its critics. Some argue that such a model risks politicizing the discipline or compromising its impartiality. But we would suggest that impartiality is not incompatible with engagement. Rather, a principled commitment to justice demands precisely the kind of responsiveness outlined above.

## 6 The Role of Bioethics in Crisis—Response to Objections

The concept of activist or advocacy‐oriented bioethics can be a topic of controversy within the field. Some critics argue that such an approach risks eroding the field's analytical rigor, compromising neutrality, or transforming it into a vehicle for political ideology. These objections warrant serious consideration, not because they undermine the need for moral responsiveness, but because they compel bioethicists to clarify what principled engagement entails.

In *Ethicists and Activists*, Kaebnick explores this tension, asking whether ethicists should remain detached analysts or take active stances in public life. While acknowledging that activism has a place in bioethics, he cautions that engagement must be rooted in thoughtful reasoning rather than driven by social or political pressure. Ethical reasoning, he argues, should guide action, not the reverse [71].

In our view, the situation in Gaza—even before October 2023 ‐ has long warranted the sort of deliberation Kaebnick calls for. Yet the picture Kaebnick paints underestimates the profound moral obligation that the suffering in Gaza places on bioethicists while simultaneously presenting a positivist notion of “objectivity.” This approach appears to assume that moral reflection can occur in a vacuum, independent of the socio‐political contexts in which bioethics operates. It neglects how power dynamics and systemic inequities, including political interests, economic structures, and cultural hegemonies, shape what is considered ethical and whose suffering is acknowledged or ignored. Such an approach perpetuates inaction, prioritizing theoretical detachment over the moral imperative to intervene in the face of grave human rights violations. A more responsible stance would adopt a reflexive, context‐sensitive approach that recognizes the partial and situated nature of crises.

Therefore, although we sympathize with Kaebnick's view that ethics must be based on careful consideration, we argue that this should not be misconstrued as an invitation to remain silent. The situation in Gaza illustrates how silence, even when framed as neutrality, can serve as a form of epistemic positioning. What bioethics fails to acknowledge often reflects what it is institutionally positioned not to see [53]. In Gaza, this silence has marginalized Palestinian suffering and excluded it from the moral imagination and gaze of the field.

Returning to Udo Schüklenk's editorial, we acknowledge his view that not all moral judgments on war fall within the scope of “bioethics expertise.” He rightly emphasizes that healthcare‐specific questions, such as the ethics of treating enemy combatants or protecting hospital neutrality, are within the field's core [55]. But we challenge the idea that broader questions of structural violence and racialized harm are external to bioethical concern. Bioethics^3^ is deeply embedded in the institutions, policies, and systems that shape health. To pretend otherwise is to adopt an artificially narrow conception of the field—one that risks making it morally irrelevant.

This narrowing is not only philosophically untenable but historically inconsistent. Bioethics has often engaged with political and structural injustice: from its critique of the Tuskegee Syphilis Study to debates on structural racism in US healthcare [72], or its advocacy for human rights in global health governance [73]. In these cases, moral clarity and political implication coexisted. The idea that bioethics must now “stay in its lane” is not consistent with its own legacy.

Furthermore, the objection that political engagement compromises impartiality also assumes that the existing moral landscape is neutral. Advocacy, in this sense, is not about adopting a political ideology; it is about refusing to ignore systematic dehumanization when it occurs under the gaze of the world. Thus, this article is not calling for simplistic alignment with one side in every conflict. It is a call for context‐sensitive moral engagement, grounded in widely held ethical principles such as non‐maleficence, justice, and the protection of health and life. Gaza is not a test of whether bioethicists are politically aligned with Palestine; it is a test of whether the field is willing to speak out when health infrastructure is deliberately destroyed, when medical workers are killed or imprisoned, and when access to care becomes a matter of political permission.

In sum, objections to activist bioethics raise important questions about the scope, authority, and responsibilities of the field. But these questions cannot justify withdrawal in the face of colossal suffering. Bioethics will remain legitimate only if it can act not as a spectator to moral collapse, but as a participant in efforts to resist it.

## 7 Conclusions

The near‐total destruction of Gaza by Israel has compelled many academics to think carefully about their personal and professional moral obligations. In this paper we have argued that bioethics as a discipline has so far failed to deliver on its moral duty to condemn the destruction of life and health in Gaza, and in doing so has undermined its own credibility and relevance. When a discipline that professes to center human dignity and justice remains largely silent in the face of widespread, systemically enabled harm, it raises questions about whether its scholars, institutions, and normative frameworks are capable of responding to real‐world ethical emergencies.

This article uses the Gaza war as a focal point to examine institutional caution, selective neutrality, and moral engagement. It argues that the stance taken by most bioethicists and bioethics institutions is not “neutral” in any virtuous sense, but is shaped by geopolitical pressures, reputational anxieties, and deeper epistemic structures that determine whose suffering is recognized, named, or engaged with. The reality that some crises provoke swift ethical responses while others are met with silence is itself an ethical phenomenon that warrants critical scrutiny.

The credibility of the field rests on its moral responsiveness to the destruction of health and life. This responsiveness need not be ideological. It can take multiple forms: speaking out against violations of medical neutrality, creating platforms for affected voices, and supporting displaced scholars and practitioners. Bioethics must move toward an orientation that is more globally attuned, justice‐driven, and inclusive of the political conditions that shape and determine health. It must expand its view of what counts as an ethical problem, recognizing that war, occupation, and racialized dispossession are not external to health, but constitutive of it. In doing so, bioethics does not need to abandon rigor or fall into partisanship. It simply needs to affirm its foundational principles beyond the Western‐centric world and worldview.

## Endnotes

1 Approximately 7%–9% of the total population in the Gaza Strip, by July 2024.

2 While it is plausible that they submitted the original paper before October 2023, there was already plenty to draw on with regard to Israel's record of wrongdoing in Palestine, and they would have had an opportunity in the revision stages to include updated material on more recent events. Indeed, the access dates on their references suggest that sources from 2024 were used.

3 It is important to clarify that bioethical engagement with wars, conflicts, and genocides does not mean applying the principles of biomedical ethics directly to warfare, as critics might suggest. Rather, it means applying these principles to the ethical responsibilities of bioethicists and the institutional frameworks of bioethics itself. As bioethics has expanded to address ethical issues beyond the clinic—such as global health disparities, environmental ethics, and pandemics—so too must it engage with the ethical consequences of war, particularly where they intersect with medical ethics and human dignity.
